# Structure of the DNA Duplex d(ATTAAT)_2_ with Hoogsteen Hydrogen Bonds

**DOI:** 10.1371/journal.pone.0120241

**Published:** 2015-03-17

**Authors:** Francisco J. Acosta-Reyes, Elida Alechaga, Juan A. Subirana, J. Lourdes Campos

**Affiliations:** Universitat Politècnica de Catalunya, Departament d’Enginyeria Química, Diagonal 647, E-08028, Barcelona, Spain; Northeastern University, UNITED STATES

## Abstract

The traditional Watson-Crick base pairs in DNA may occasionally adopt a Hoogsteen conformation, with a different organization of hydrogen bonds. Previous crystal structures have shown that the Hoogsteen conformation is favored in alternating AT sequences of DNA. Here we present new data for a different sequence, d(ATTAAT)_2_, which is also found in the Hoogsteen conformation. Thus we demonstrate that other all-AT sequences of DNA with a different sequence may be found in the Hoogsteen conformation. We conclude that any all-AT sequence might acquire this conformation under appropriate conditions. We also compare the detailed features of DNA in either the Hoogsteen or Watson-Crick conformations.

## Introduction

The DNA double helix is usually stabilized by Watson-Crick (W-C) hydrogen bonds between the bases. Occasionally a different type of structure is found: the adenine bases may rotate 180° and form a different set of hydrogen bonds, first demonstrated by Hoogsteen using X-ray crystallography[1]. Hoogsteen hydrogen bonds may also occur in C·G base pairs, but they are less frequently found, since they require protonation of cytosine. In general Hoogsteen hydrogen bonds are only observed at a low frequency as transient structures in canonical duplex DNA [2]. They have been also described in the Y family of polymerases [3], in particular in the interaction of polymerase **ι** with DNA [4–6], a polymerase which is able to replicate DNA through a lesion.

When a double helix is built with A·T Hoogsteen hydrogen bonds, an antiparallel double helix is formed, which has an appearance very similar to the standard B form of DNA [7]. The main intrinsic change is the rotation of the glycosidic bond by 180°, so that the nucleoside conformation of adenine changes from *anti* to *syn*. As a result a new set of hydrogen bonds is formed: the N3 atom of adenine is moved from the minor to the major groove. The minor groove is narrower and looses hydrogen bonding capacity. A clear signature of the Hoogsteen form is the 2 Å shortening of the sugar C1’-C1’ distances across each base pair, which is clearly apparent in the structures determined by X-ray crystallography.

In this paper we present a continuous Hoogsteen duplex with a non-alternating sequence, d(ATTAAT), which contains AA/TT base steps. In previous crystallographic studies we only found the Hoogsteen conformation in alternating AT DNA sequences [7–9], whereas most mixed sequence all-AT DNAs only gave W-C structures [10]. Our results indicate that it should be possible to find long stretches of all-AT DNA in the Hoogsteen conformation under appropriate conditions.

The availability of these new data, together with those previously available, allows us to study in more detail the structural features of Hoogsteen DNA, which may help to determine its eventual biological role. It is known that AT base pairs predominate in the non-coding regions of the genome [11]. Thus Hoogsteen DNA may play a structural role in the genome, a question which we will analyze in this paper. We will also compare in detail the main features of all-AT DNA in either the Hoogsteen or W-C conformations.

## Materials and Methods

The oligonucleotide d(ATTAAT) was synthesized at the Pasteur Institute as the ammonium salt on an automatic synthesizer by the phosphoramidite method. It was purified by gel filtration and reverse–phase HPLC.

Hexagonal prism shaped crystals of the oligonucleotide were grown by vapor diffusion at 286 K, using the hanging-drop method. The concentrations in the drop were as follow: 0.8mM oligonucleotide, 20mM sodium cacodylate buffer (pH = 6.5), 3mM spermine, 100mM N-ethylaniline hydrochloride and 5% methyl-pentanediol (MPD), equilibrated against a reservoir with 30% MPD. Crystals appeared after 2 months.

Crystals were flash-cooled in liquid nitrogen (100K) and stored until data collection. The MPD in the crystallization medium works as a cryoprotectant.

X ray diffraction data were collected in the BM16 line at ESRF, with a resolution up to a 3 Å, at 100K, λ = 0.9761 Å and φ = 1.5°. Data processing, indexing and scaling was done with the programs XDS and Xscale [12]. The structure was solved by molecular replacement with the Phaser program [13] with two and a half DNA duplexes in the asymmetric unit. Restrained maximum likelihood refinement was done with the program Refmac [14]. Final crystallographic data is reported in Table 1. Details of Structure resolution and Refinement are given in the Supporting Information. Coordinates were deposited in the PDB data file, code 2QS6.

**Table 1:** Crystallographic data for d(ATTAAT).

Wavelength (Å)	0.9761
Resolution range (Å)	33.14–3.13 (3.32–3.13)
Space group	*C* 1 2 1
Unit cell (*a*, *b*, *c*, α, β, γ)	24.76, 42.90, 99.42, 90.0°, 90.04°, 90.0°
Total reflections	7048 (7048)
Unique reflections	1 888 (279)
Multiplicity	3.7 (3.8)
Completeness (%)	99.6 (99.3)
Mean I/sigma(I)	7.48 (1.88)
Wilson B-factor	141.89
*R*_meas_	0.125 (0.254)
CC_1/2_	0.985 (0.96)
CC*	0.996 (0.99)
*R*_work_ / *R*_free_	0.146 / 0.199
Number of non-hydrogen atoms	600
RMS(bonds)	0.009
RMS(angles)	1.25
Average B-factor	138.5

Details of Structure resolution and Refinement are given in the Supporting Information.

## Results

### Structural features of d(ATTAAT)

The presence of N-ethyl-aniline hydrochloride in the crystallization conditions was suggested by the observation that the Hoogsteen duplexes previously studied [7–9] had thymine bases in the minor groove: the hydrophobic ethyl-aniline might play a similar role. However we did not detect any trace of ethyl-aniline in the minor groove. Also crystallization trials with this solvent and other all-AT DNA sequences did not yield any useful crystals.

**Fig 1:**
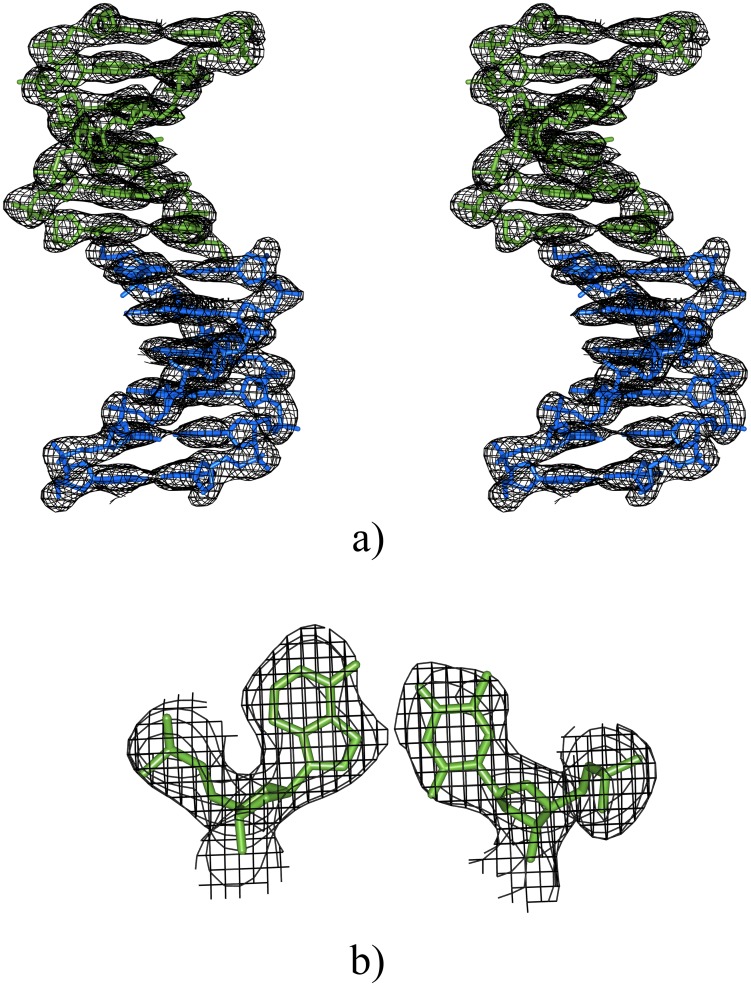
Electron density maps of d(ATTAAT)_2_. 2Fo-Fc electron density maps at 1σ level, with Refmac5 map sharpening [24]: (a) stereo view of two duplexes. All base pairs are in the Hoogsteen conformation. The shift between the two duplexes is very clear at the center of the column. It is shown in detail in Fig. 4; (b) one of the central base pairs. Other base pairs have a similar map.

In the crystal the molecules are organized as columns of stacked duplexes, which are packed in a pseudo-hexagonal lattice. Attempts to solve the structure in the W-C conformation were not successful, as it is described in the Supporting Information. Views of the structure are presented in Fig. 1 and in the Supporting Information (see S1 Fig.). The electron density map of base pairs could be matched with the bases in the Hoogsteen conformation. Furthermore the average distance between the sugar C1’ atoms was short as expected. One of the most significant differences between W-C and Hoogsteen base pairs is precisely the shorter value of the C1’-C1’ distance in each base pair, as shown in Table 2.

**Table 2:** Comparison of conformational parameters.

(PDB-ID)	Hoogsteen				Watson-Crick	
	ATTAAT (4U9M)	ATATAT [7] (1RSB)	ATATATCT [9] (2QS6)	ATATATATAT [15] (3EY0)		AAATATTT [16] (2A2T)
C1'-C1' [Å]	8.5	8.2	8.1	8.5	10.4	10.6
ω_i_ (AT)	29.5	33.7	31.0	—	31.1	34.0
ω_i_ (TA)	34.8	34.7	36.0	—	41.4	38.5
ω_i_ (AA/TT)	38.1	—	—	—	—	37.0
ω_T_ (TA)	42.4	43.6	40.5	46.9	-13.0	-25.2

References are given in brackets. ω_i_ is the average twist of the base steps and ω_T_ is the average twist of the virtual base step between neighbor duplexes in a column (Calculated with 3DNA[25] from the C1’-C1’ virtual bonds).

### Internal features of Hoogsteen duplexes

The main features of the Hoogsteen conformation were already analyzed in detail in a previous publication [7]. It was found that the overall features of the double helix are strikingly similar in both, in spite of the shorter C1’-C1’ distance in the Hoogsteen conformation. The minor groove is narrow, but similar to all-AT W-C duplexes, due to a different position of the helical axis.

**Fig 2:**
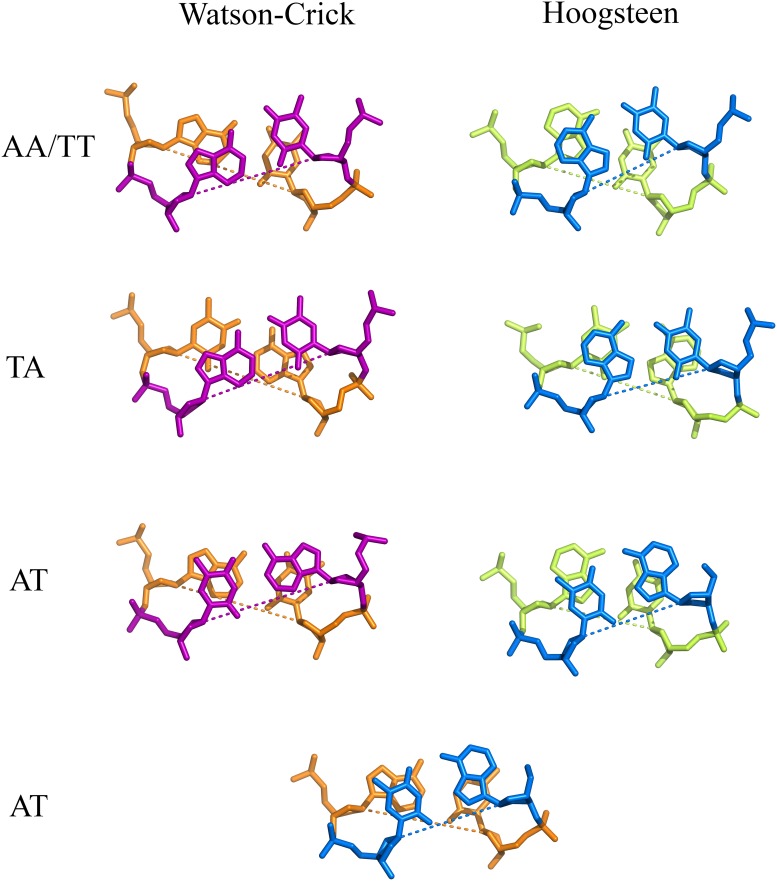
Base step stacking comparison in the Watson-Crick and Hoogsteen conformations. It is similar in the AA and AT base steps, including the unique W-C/Hoogsteen case found in the 3EY0 structure [15]. The TA base step is different in each case, with poor stacking in the Watson-Crick structures: it is shown in greater detail in the supporting information (see S2 Fig.). The Hoogsteen base pairs are shown in blue/green and the Watson-Crick base pairs in orange/purple in this and in the following figures. The dashed lines indicate virtual C1’—C1’ bonds.

The availability of the new Hoogsteen structure for d(ATTAAT)_2_, together with other complete [7–9] or partial [15] structures, allows a more detailed comparison of the Hoogsteen and W-C conformations, as presented in Fig. 2 and in Table 2. It is apparent that the AT and AA/TT base steps have similar values of the ω_i_ twist angle in both conformations. Stacking is also similar, with a poor stacking of thymines in both AA/TT base steps (Fig. 2). In the case of the AT base step, the Hoogsteen/W-C stacking found in one case [15] is also similar.

The main conformational difference between the two conformations is found in the TA base step, which presents significantly different ω_i_ twist values (Table 2). Also stacking is very poor in the W-C case (Fig. 2). This difference between both structures has been confirmed in all available cases, as shown in the supporting information (see S2 Fig.).

### Stacking interactions between all-AT duplexes

**Fig 3:**
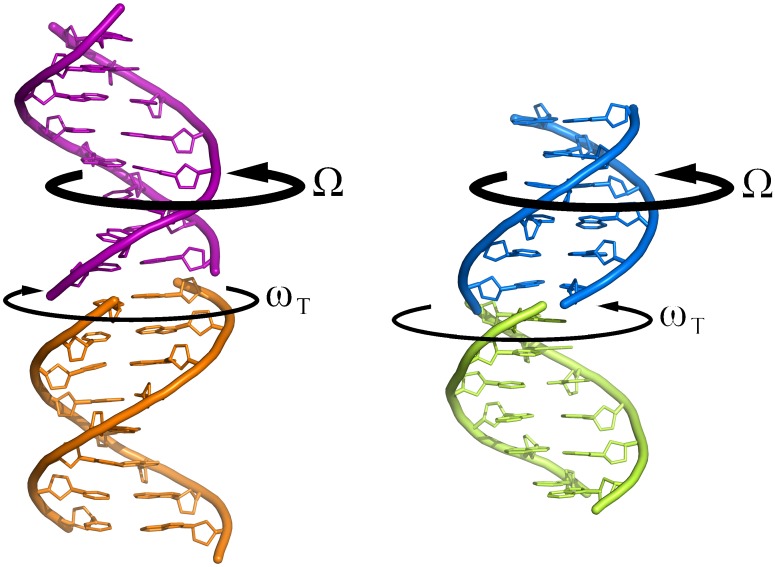
Comparison of the d(AAATATTT)_2_ Watson-Crick structure and the d(ATTAAT)_2_ Hoogsteen structure. The overall twist Ω and the virtual base step twist ω_T_ between stacked duplexes are indicated. In the Hoogsteen case the double helix is practically continuous throughout the column of duplexes.

DNA duplexes in most all-AT crystals form continuous columns of stacked double helices (Fig. 3). The stacked duplexes form a pseudo-continuous double helix. In this configuration the overall relative twist Ω of an oligonucleotide duplex with respect to its nearest neighbor in the column is defined as:

$$\Omega =\omega_{T}+\sum_{i=1}^{i=5}\omega_{i}$$ (1)

Where ω_i_ correspond to the twist angle between adjacent base pairs in the oligonucleotide and ω_T_ correspond to the rotational setting angle of the virtual base step between stacked duplexes. In all Hoogsteen structures the value of ω_T_ between neighbor duplexes is similar and close to 40°, only somewhat larger that the twist angle in the base steps inside the duplexes, as shown in Table 2. As a result the columns of duplexes form a perfect pseudo-continuous helix, as shown in Fig. 3. This behavior is completely different from what is found in all-AT duplexes with Watson-Crick base pairs [10, 16], which also crystallize in columns, but with a left handed ω_T_ negative angle, usually around -25°. Several examples are given in Table 2. The values for d(ATATATATAT) deviate, since in this structure the columns of duplexes are not straight, but form a coiled-coil [15].

**Fig 4:**
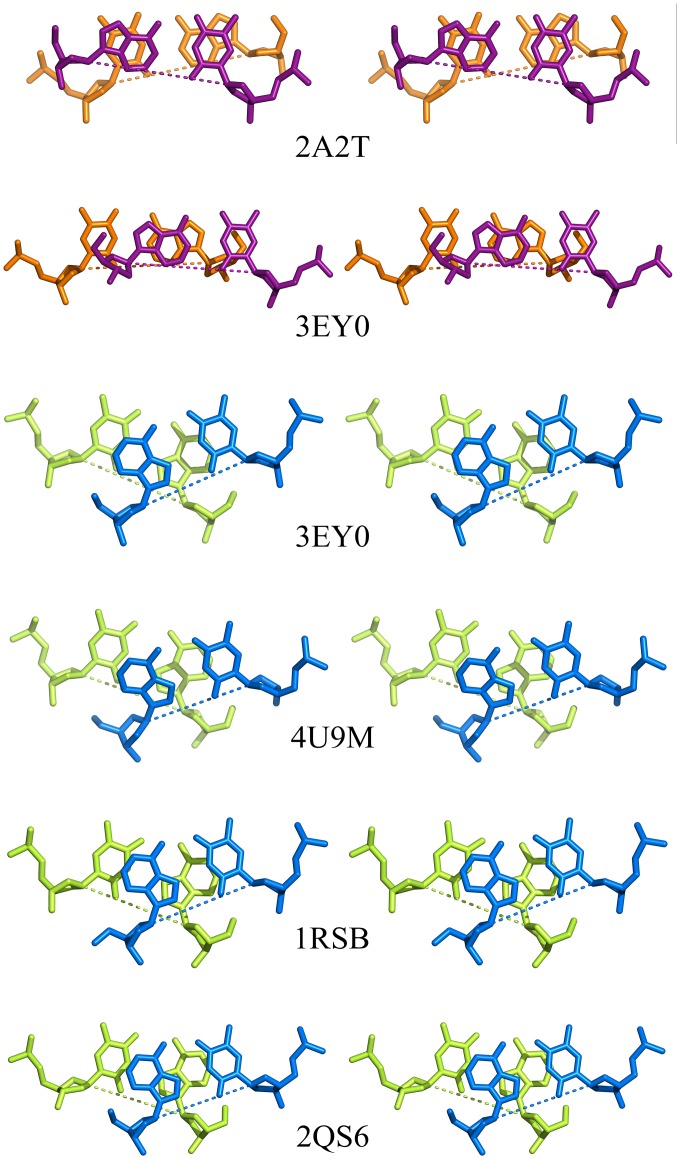
Stereo views of the virtual TA base step between neighbor duplexes in a column in different Watson-Crick and Hoogsteen structures. In all Hoogsteen cases the base pairs show a similar slide and a practically identical twist angle, close to 40° (Table 2). In the Watson-Crick case ω_T_ is negative. The dashed lines indicate virtual C1’—C1’ bonds. The PDB codes are indicated.

In fact the most striking difference between the W-C and Hoogsteen structures resides in the virtual TA base step between neighbor duplexes, which has a different sign in either case, as mentioned above. In this way base stacking is improved in W-C duplexes, as it is clearly apparent in Fig. 4. In the Hoogsteen case only adenine stacking is favored, whereas thymines are left poorly stacked. A significant slide of both base pairs is also apparent.

## Discussion

A significant feature of the present structure is the absence of any ligand in the minor groove. In our previous studies [7–9] we had found that extra-helical bases, mainly thymines, were present and might contribute to the stabilization of Hoogsteen DNA. The present structure is stable by itself and does not require any additional ligands, but it is not clear why it crystallizes in the Hoogsteen form. No specific interactions with other molecules which might stabilize the crystal structure have been detected. In particular no trace of ethyl-aniline is apparent in the crystal structure, although it might help to stabilize the Hoogsteen form. Since the energetic difference between the Hoogsteen and W-C forms is rather small [17, 18], it is likely that a combination of crystallization conditions, oligonucleotide length and sequence favored the Hoogsteen form in this particular case.

In conclusion, the structure that we have presented here demonstrates that in principle any all-AT sequence of DNA should be capable of adopting the Hoogsteen conformation. Our work complements the results of Nicolova et al. [2, 19], who have shown that transient Hoogdteen base pairs are frequent in canonical duplex DNA. Such transient base pairs may be a nucleation point for continuous Hoogsteen duplexes, such as those described here and elsewhere [7–9]. Occasional base pairs are also found in some complexes of DNA with drugs and proteins, as reviewed by Nicolova et al [20]. It is also likely that some regions of the genome may have this conformation “in vivo” under appropriate conditions, since the transition between both forms has a very low energetic cost. The tendency to accommodate extra-helical bases in the minor groove [7–9] may help to form DNA crosslinks during meiosis [21] or in the compact territories of gene-poor chromosomes in the nucleus [22]. Furthermore the formation of continuous helices (Fig. 3), even when there is a break in the chain, may help in the repair of double strand breaks, in DNA recombination, in the action of topoisomerases, etc. We should note that base-stacking is an important contribution to the stability of the double helix [23].

## Supporting Information

S1 Fig Crystal packing. The organization of duplexes in the crystal in pseudo hexagonal packing is shown at the left. The unit cell is indicated. The column at the right is formed by five stacked duplexes, which correspond to two asymmetric units.
(TIF)

S2 Fig Stereo views of all internal TA base steps. Comparison of superposed steps in different Watson-Crick (orange/purple) and Hoogsteen (green/blue) structures. The TA step shows better stacking for Hoogsteen than WC. The PDB codes are indicated.
(TIF)

S3 Fig ω_T_ angle in WC and Hoogsteen. Comparison of two columns of five standard B-DNA duplexes with Watson-Crick base pairing and ω_T_ = -26° on the left and Hoogteen with ω_T_ = 42° on the right. Note the very different overall geometry of the columns.
(TIF)

S4 Fig Pseudo hexagonal symmetry P1 related to C2. (TIF)

S1 Table The five twin operators found for the C2 space group and the six twin domains and its fraction as refined in Refmac5. (PDF)

S1 File Structure solution and refinement. (PDF)
