# Supplementary figures and images for: Structure of the DNA Duplex d(ATTAAT)2 with Hoogsteen Hydrogen Bonds
Source: PLoS One. 2015 Mar 17;10(3):e0120241. doi: 10.1371/journal.pone.0120241 (PMC4363561; doi:10.1371/journal.pone.0120241)

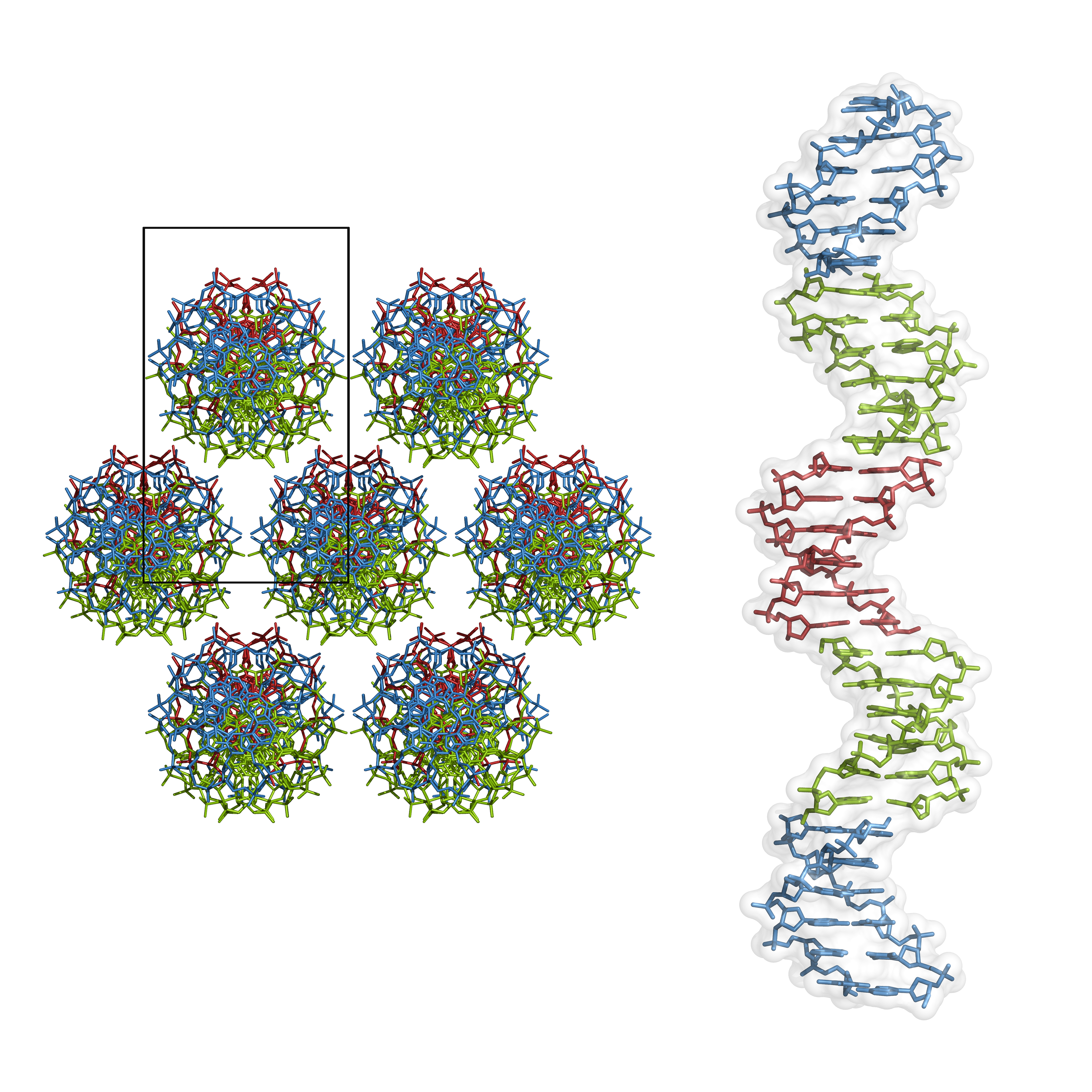

Supplement: S1 Fig — The organization of duplexes in the crystal in pseudo hexagonal packing is shown at the left. The unit cell is indicated. The column at the right is formed by five stacked duplexes, which correspond to two asymmetric units. (TIF) [file pone.0120241.s001.tif]

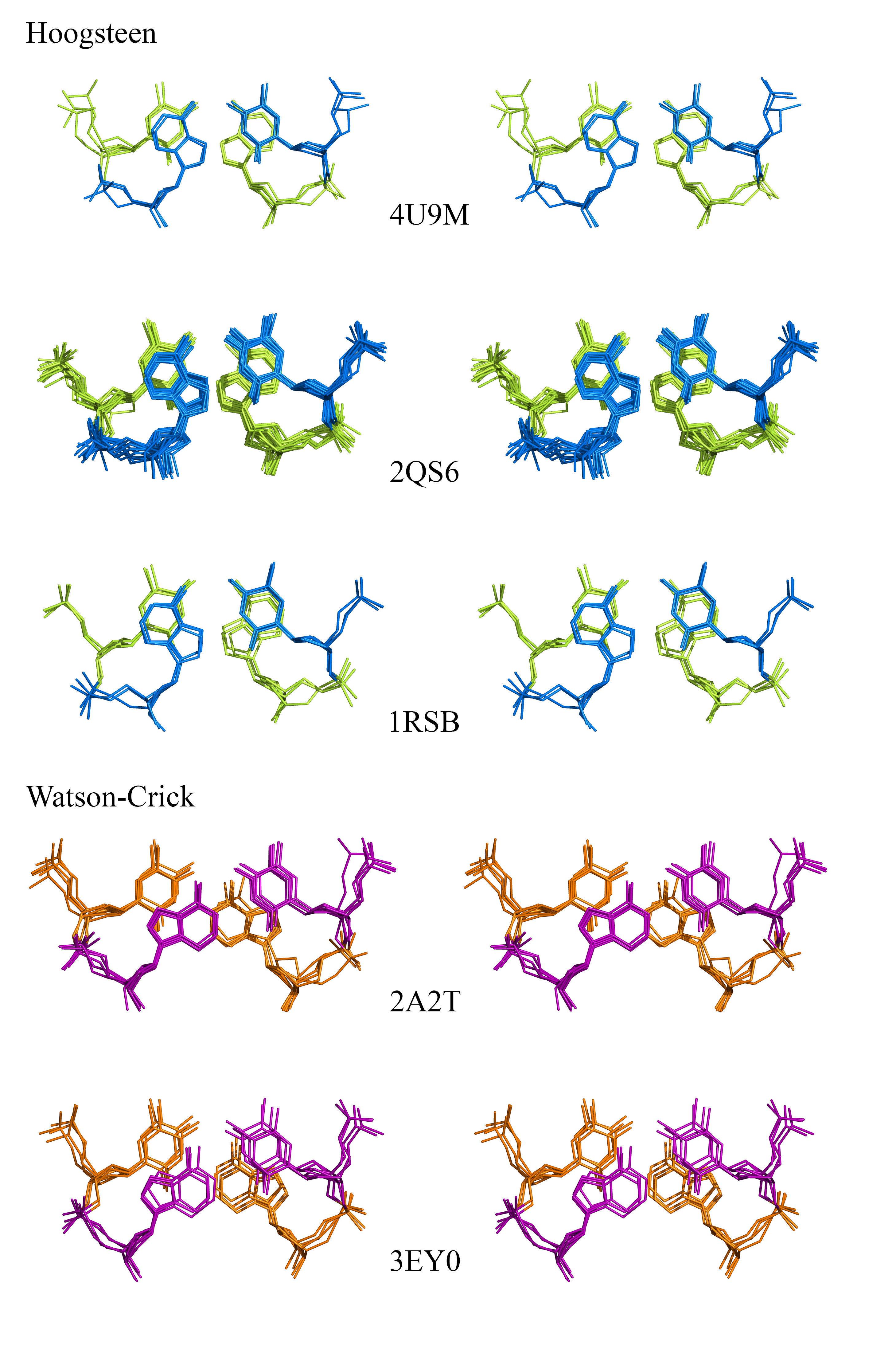

Supplement: S2 Fig — Comparison of superposed steps in different Watson-Crick (orange/purple) and Hoogsteen (green/blue) structures. The TA step shows better stacking for Hoogsteen than WC. The PDB codes are indicated. (TIF) [file pone.0120241.s002.tif]

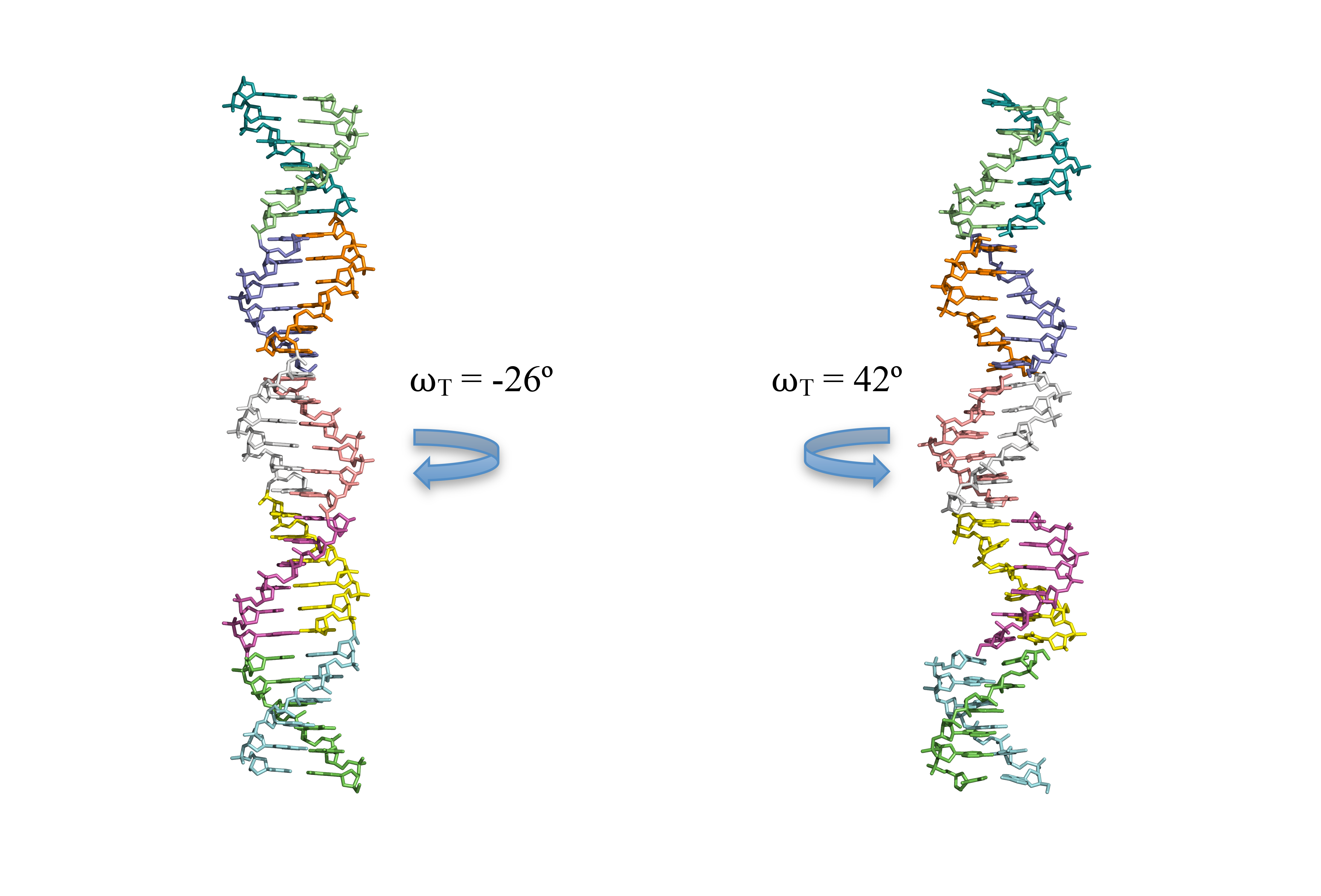

Supplement: S3 Fig — Comparison of two columns of five standard B-DNA duplexes with Watson-Crick base pairing and ωT = -26° on the left and Hoogteen with ωT = 42° on the right. Note the very different overall geometry of the columns. (TIF) [file pone.0120241.s003.tif]

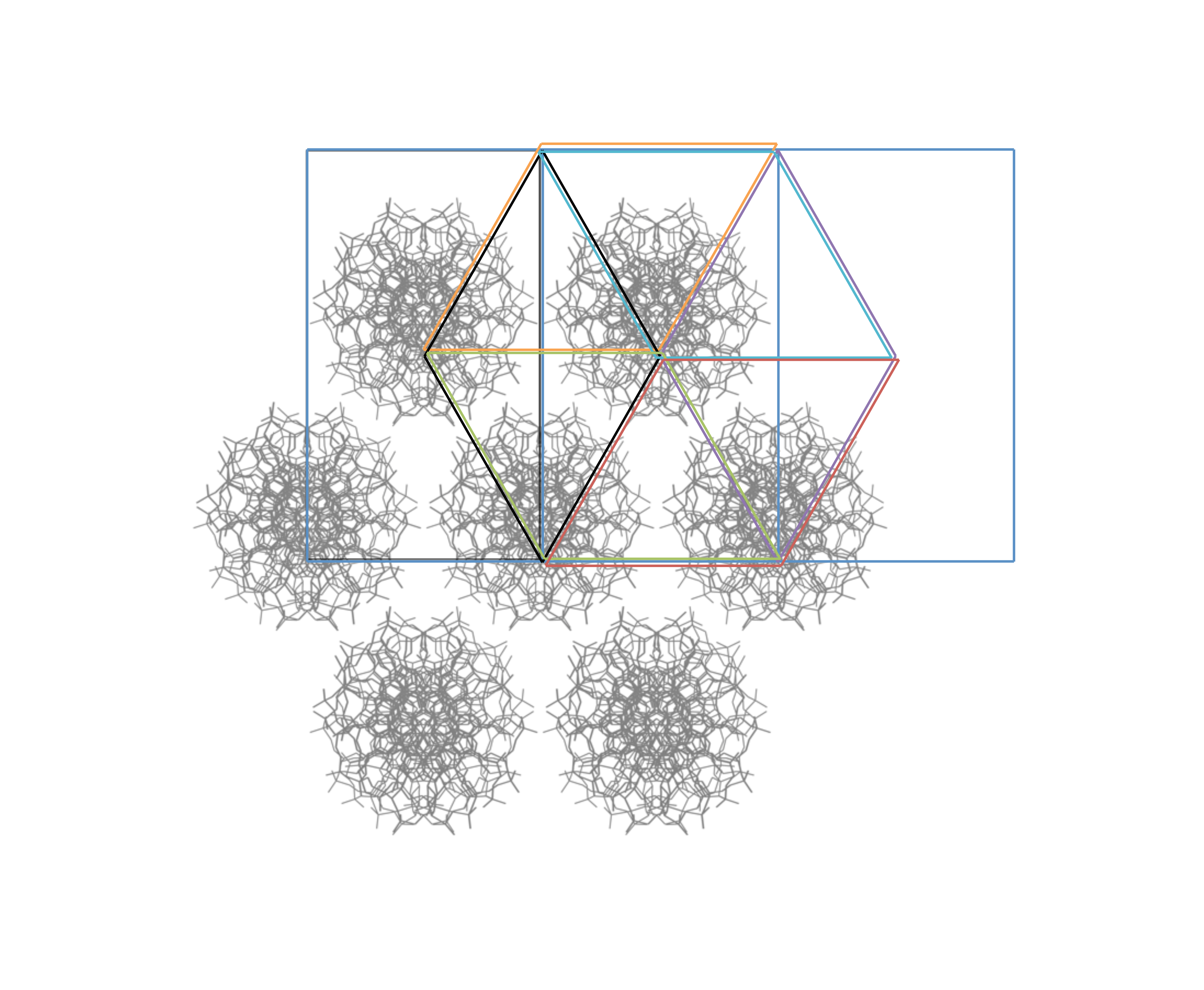

Supplement: S4 Fig — (TIF) [file pone.0120241.s004.tif]
