# Supplementary material for: Structure of the DNA Duplex d(ATTAAT)2 with Hoogsteen Hydrogen Bonds
Source: PLoS One. 2015 Mar 17;10(3):e0120241. doi: 10.1371/journal.pone.0120241 (PMC4363561; doi:10.1371/journal.pone.0120241)
Supplement: S1 Table — (PDF) [file pone.0120241.s005.pdf]

S1 Table. The five twin operators found for the C2 space group and the six twin domains and its fraction as refined in Refmac5.

Domain	Operator	Fraction
**1**	H, K, L	0.184
**2**	1/2H+1/2K, -3/2H+1/2K, L	0.156
**3**	1/2H-1/2K, 3/2H+1/2K, L	0.147
**4**	-H, -K, L	0.168
**5**	-1/2H+1/2K, -3/2H-1/2K, L	0.185
**6**	-1/2H-1/2K, 3/2H-1/2K, L	0.159
