# Supplementary material for: Structure of the DNA Duplex d(ATTAAT)2 with Hoogsteen Hydrogen Bonds
Source: PLoS One. 2015 Mar 17;10(3):e0120241. doi: 10.1371/journal.pone.0120241 (PMC4363561; doi:10.1371/journal.pone.0120241)
Supplement: S1 File — (PDF) [file pone.0120241.s006.pdf]

S1 File. Structure Solution and Refinement

X ray diffraction data were collected in the BM16 line at ESRF, with a resolution up to a 3 Å, at 100K, λ = 0.9761 Å and φ = 1.5º. Data processing, indexing and scaling was done with the programs XDS and Xscale [1]. Data indexing and space group identification with program pointless [2] indicated a hexagonal space group *P*622. It was impossible to solve the structure in either hexagonal or trigonal space groups: molecular replacement did not match any possible arrangement.

Using as search model a standard B-DNA duplex, it was tested by molecular replacement in Bravais lattices *hP*, *oC*, *mP*, *mC* and *aP* (*P*6*, *P*3*, *C*222, *C*2, *P*2* and P1) with no success. A pseudo-translation was detected, but using the corresponding translation vector it did not help in molecular replacement. Although in some cases molecules were placed as expected in parallel columns, the high values in Rfactor (Rwork and Rfree) and the broken electron density in 2Fo-Fc map indicated it was not a real solution.

The structure was finally solved in the space group *P*1 (24.72 24.72 99.35 90.02º 90.02º 119.95º) by molecular replacement with the Phaser program [3]. A model of 5 duplexes in a column was used. It was based on the stacking diffraction of base pairs, which showed a rise of 3.3Å. The proper helical parameters and rotational setting angle ω_T were used.

In a first attempt to solve the structure we built two models of B-DNA with Watson-Crick base pairing, one with standard twist ω_i=36º, rise 3.31Å and ω_T = -36º, and the other with twist ω_i=34º and ω_T = -26º (S3 Fig.) with no success when used as search model for molecular replacement. In both cases the electron density was fragmented. It was only possible to solve the structure using a model constructed with Hoogsteen base pairing based on PDB-IDs 2QS6 and 1RSB with a column of 5 duplexes with twist 35º and ω_T = 41 (S3 Fig.). Finally the solution was translated to a higher symmetry space group *C*121.

The final space group and unit cell of the structure is *C*121 (*a, b, c, α, β, γ*: 24.76, 42.90, 99.42, 90.00º, 90.04º, 90.00º) with a resolution up to 3.13 Å. The Patterson function of the data showed pseudo translation symmetry with an off-origin peak of 36% of the origin peak in the coordinates (0, 0, 0.43). A contribution to this pseudo translation comes from vertical displacements of neighbor columns of duplexes and rotational shifts.

Refinement was done with the program Refmac5 [4]. After several cycles of maximum-likehood restrained refinement, we added H-bond distance restrains which were removed in the last round of refinement. In the final stage twin refinement was used, and the $R_{factors}$ dropped considerably to a final value of R_{work}=14.6 and R_{free}=19.99. From L test for acentric data with a mean |L|=0.472 (untwined=0.5, perfect twin=0.375) and mean L^2=0.304 (untwined=0.333, perfect twin=0.2) there was little possibility of suspected twining based on the intensity statistics. In spite of the results of twining tests, twining is very likely: detection of twinning is very difficult due to the presence of pseudo translational or pseudo rotational non-crystallographic symmetry parallel to the twinning axis. Also the presence of the apparent higher symmetry as a hexagonal space group (S4 Fig.) and the drop of R-factors with twin refinement, confirmed the presence of a pseudo-merohedral twinning with 5 twin operators (Table S1). From Wilson Plot the estimated B factor was 141, after refinement the final average B factor was 138.5, the ADP statistics of the distribution of B factor, in all atoms, does not show values over 4σ, indicating no suspicious distribution in B values. The final structure was compared against a structure refined with B factors restrained to about 25 (R_{work}=0.20 and R_{free}=0.22), with no discrepancies between them. From this result we decided not to restrain B factors, as there were no significant changes in the structure. Electron density maps were improved by using map sharpening [5] in Refmac5. Figures were made using programs CCP4MG [6] and Pymol [7].

References

1. Kabsch W. XDS. Acta Crystallogr D Biol Crystallogr. 2010;66: 125-132.
2. Evans P. Scaling and assessment of data quality. Acta Crystallogr D Biol Crystallogr. 2006;62: 72-82.
3. McCoy AJ, Grosse-Kunstleve RW, Adams PD, Winn MD, Storoni LC, Read RJ, et al. Phaser crystallographic software. J Appl Crystallogr. 2007;40: 658-674.
4. Murshudov GN, Skubák P, Lebedev AA, Pannu NS, Steiner RA, Nicholls RA, et al. REFMAC5 for the refinement of macromolecular crystal structures. Acta Crystallogr D Biol Crystallogr. 2011;67: 355-367.
5. Nicholls RA, Long F and Murshudov GN. Low-resolution refinement tools in REFMAC5. Acta Crystallogr D Biol Crystallogr. 2012;68: 404-417.
6. McNicholas S, Potterton E, Wilson KS and Noble ME. Presenting your structures: the CCP4mg molecular-graphics software. Acta Crystallogr D Biol Crystallogr. 2011;67: 386-394.
7. Schrodinger LLC. The PyMOL Molecular Graphics System, Version 1.3r1. 2010.
